# Protective Efficacy Induced by the Common *Eimeria* Antigen Elongation Factor 2 against Challenge with Three *Eimeria* Species in Chickens

**DOI:** 10.3390/vaccines12010018

**Published:** 2023-12-22

**Authors:** Yuxuan Mi, Wenxi Ding, Lixin Xu, Mingmin Lu, Ruofeng Yan, Xiangrui Li, Xiaokai Song

**Affiliations:** MOE Joint International Research Laboratory of Animal Health and Food Safety, College of Veterinary Medicine, Nanjing Agricultural University, Nanjing 210095, China; 2021107064@stu.njau.edu.cn (Y.M.); 2018807149@njau.edu.cn (W.D.); xulixin@njau.edu.cn (L.X.); mingmin.lu@njau.edu.cn (M.L.); yanruofeng@njau.edu.cn (R.Y.); lixiangrui@njau.edu.cn (X.L.)

**Keywords:** avian coccidiosis, common antigen, EF2, multivalent vaccine, co-infection

## Abstract

Avian coccidiosis arises from co-infection involving multiple *Eimeria* species, which could give rise to substantial economic losses in the global poultry industry. As a result, multivalent anticoccidial vaccines containing common *Eimeria* antigens offer considerable promise for controlling co-infection in clinical practice. In our previous study, Elongation factor 2 (EF2) was deemed as an immunogenic common antigen across various *Eimeria* species. This current investigation aimed to further assess the immunogenicity and protective efficacy of EF2 in recombinant subunit vaccine format against three *Eimeria* species. The EF2 gene cloned from *Eimeria maxima* (*E. maxima*) cDNA was designated as EF2 of *E. maxima* (EmEF2). The immunogenicity of the recombinant protein EmEF2 (rEmEF2) was assessed through Western blot analysis. The evaluation of the vaccine-induced immune response encompassed the determination of T lymphocyte subset proportions, cytokine mRNA transcription levels, and specific IgY concentrations in rEmEF2-vaccinated chickens using flow cytometry, quantitative real-time PCR (qPCR), and indirect enzyme-linked immunosorbent assay (ELISA). Subsequently, the protective efficacy of rEmEF2 was evaluated through vaccination and challenge experiments. The findings demonstrated that rEmEF2 was effectively recognized by the His-tag monoclonal antibody and *E. maxima* chicken antiserum. Vaccination with rEmEF2 increased the proportions of CD4^+^ and CD8^+^ T lymphocytes, elevated IL-4 and IFN-γ mRNA transcription levels, and enhanced IgY antibody levels compared to the control groups. Moreover, compared to the control groups, vaccination with rEmEF2 led to decreased weight loss, reduced oocyst outputs, and alleviated enteric lesions. Furthermore, in the rEmEF2-immunized groups, challenges with *E. maxima* and *E. acervulina* resulted in anticoccidial index (ACI) scores of 166.35 and 185.08, showing moderate-to-excellent protective efficacy. Nevertheless, challenges with *E. tenella* and mixed *Eimeria* resulted in ACI scores of 144.01 and 127.94, showing low protective efficacy. In conclusion, EmEF2, a common antigen across *Eimeria* species, demonstrated the capacity to induce a significant cellular and humoral immune response, as well as partial protection against *E. maxima*, *E. acervulina,* and *E. tenella*. These results highlight EmEF2 as a promising candidate antigen for the development of multivalent vaccines targeting mixed infections by *Eimeria* species.

## 1. Introduction

Coccidiosis in chickens, characterized by a hemorrhagic presentation, typically results in high mortality rates, subpar weight gains, and inefficient food conversion ratios. This has a profound impact on chicken production and overall welfare [1,2]. In 2016, the global economic burden caused by chicken coccidiosis arising from multiple *Eimeria* species was estimated to have surpassed GBP 10.4 billion [3,4]. Avian coccidiosis is prevalent worldwide, with an incidence ranging from 70% to 90% [5], often stemming from mixed infections involving multiple *Eimeria* species [6]. Currently, anticoccidial drugs have been the primary approach for managing avian coccidiosis [7,8]. However, the continuous occurrence of drug resistance and the presence of drug residues in poultry products are constantly escalating, which has led to the search for new approaches, such as the use of anticoccidial vaccines as a substitute for chemoprophylaxis prevention to control coccidiosis [9,10]. In recent years, vaccination with innovative vaccine types, including subunit vaccines, DNA vaccines, and live vector vaccines, has emerged as a promising strategy [11,12,13,14]. Recent research has demonstrated the successful utilization of various *Eimeria* antigens to develop neotype anticoccidial vaccines that offer effective protection [11,14,15,16,17,18,19,20]. However, the majority of these antigens are derived from single *Eimeria* species and do not fulfill the clinical requirement for controlling mixed *Eimeria* infections.

As avian coccidiosis typically arises from mixed infections involving multiple *Eimeria* species in clinical settings, identifying common proteins shared by various *Eimeria* species becomes essential for the development of multivalent vaccines against this poultry disease. Several *Eimeria* common antigens have been reported in this regard. For instance, Talebi discovered a 45 kDa immunogenic protein that was recognized by chicken antiserum against *E. maxima* and remained conserved among five *Eimeria* species [21]. A common antigen of all chicken *Eimeria* species has been clearly identified by Sasai et al., which is present on motile stages and can be recognized by chicken monoclonal antibodies against *E. acervulina* sporozoites; in addition, the presence of this antigen has been found in two closely related coccidian parasites (*Toxoplasma* and *Neospora*) [22]. In our previous studies, we successfully identified five common immunodominant antigens displaying an amino acid sequence similarity of over 93% among three *Eimeria* species (*Eimeria maxima, Eimeria acervulina,* and *Eimeria tenella*). Notably, two of these antigens have been demonstrated to provide effective protection against infections caused by these three *Eimeria* species, both individually and in combination [23,24,25]. These studies strongly underscore the potential of *Eimeria* common antigens as prospective candidate components for the development of effective, safe, and stable multivalent vaccines against avian coccidiosis in poultry.

Elongation factor 2 (EF2), a member of the GTP-binding translation elongation factor family, has garnered attention in various research domains [26,27,28,29]. It has been demonstrated to exhibit a high degree of conservation among various apicomplexan protozoa and has been proposed as a potential target for drugs or vaccine candidate antigens against protozoan diseases [30,31]. In the case of *Leishmania*, Agallou et al. reported remarkable EF2 conservation between strains, including *L. infantum*, *L. donovani*, *L. major,* and *L. braziliensis*, with amino acid sequence identities ranging from 98% to 100% [32]. Furthermore, EF2 was identified as a T cell-stimulating antigen capable of eliciting protective cellular immune responses against experimental visceral leishmaniosis [32,33,34]. Extensive analysis of 1685 clinically infected *Plasmodium* samples from 17 countries revealed the high conservation of EF2 in *Plasmodium* genomic sequences, and inhibiting EF2 at multiple stages of *Plasmodium* growth resulted in a substantial reduction in the *Plasmodium* population of up to 98% [35]. In the study of *Plasmodium*, in order to block the transmission of malaria, Dechering et al. identified drugs targeting EF2 as important candidates [36]. In our previous research, among *Eimeria* species, EF2 was deemed as a common immunodominant antigen, sharing an astonishing amino acid sequence similarity of 99% among the three *Eimeria* species studied [24]. However, the protective efficacy of EF2 against infections caused by different *Eimeria* species remained unknown. In this study, the EF2 gene of *Eimeria maxima* (EmEF2) was ligated with the prokaryotic expression vector to produce the recombinant protein EmEF2 (rEmEF2). Subsequently, we systematically assessed the cellular and humoral immune responses triggered by EF2 in the form of rEmEF2 in chickens. Finally, we evaluated the protective efficacy of rEmEF2 through vaccination and challenge experiments. These results indicate that EmEF2 may confer partial protection against multiple *Eimeria* infections and hold promise as a candidate antigen for the development of multivalent vaccines to control avian coccidiosis in practical clinical applications. It may provide new ideas for the development of multivalent vaccines against other pathogens.

## 2. Materials and Methods

### 2.1. Animals, Parasites and Antiserum

Hy-Line chickens (1-day-old) were raised under stringent condition in a coccidia-free environment in the Laboratory Animal Center of Nanjing Agricultural University. Meanwhile, chickens had unrestricted access to water and feed without any coccidiostat during the experimental period. Each chicken underwent oral infection with 1 × 10^4^ *E. acervulina*, and oocysts were collected from feces 4–7 days post-infection. Subsequently, each chicken was orally infected with 1 × 10^4^ *E. maxima* and 8 × 10^3^ *E. tenella*, and oocysts were collected from feces 5–8 days post-infection. Oocysts were collected from feces using the saturated saline floatation method. Sporulated oocysts of *E. maxima*, *E. acervulina* and *E. tenella* were stored at 4 °C in 2.5% potassium dichromate. To ensure the viability of the parasites, sporulated oocysts were propagated in chickens seven days prior to the challenge trials. All animal procedures and experiments were subject to rigorous ethical scrutiny and received approval from the Committee on Experimental Animal Welfare and Ethics of Nanjing Agricultural University (Approval number: PTA 2020001). The non-infected chicken serum and *E. maxima* chicken antiserum were provided by our lab [16] and used for subsequent Western blot analysis.

### 2.2. Cloning of EmEF2 and Recombinant Plasmid Construction of pET-32a-EmEF2

Total RNA extraction from 1 × 10^8^ *E. maxima* was carried out utilizing a Total RNA Extraction Kit (Omega Bio-Tek, Norcross, GA, USA). Then, using the HiScript III Q RT SuperMix for qPCR (+gDNA wiper) (Vazyme Biotech, Nanjing, China), the extracted total RNA from *E. maxima* served as the starting material for cDNA synthesis. The reverse transcription steps were as follows: 1 µg of total RNA and 4 µL of 4 × gDNA wiper Mix were added to a RNase-free EP tube, followed by RNase-free water to 16 µL and 42 °C for 2 min. We then added 4 µL of 5 × HiScript III qRT SuperMix to the tube at 37 °C for 15 min, then 85 °C for 5 s. The cDNA was used for the subsequent experiments. Restriction enzyme-anchored primers were designed and guided by the sequence of *E. maxima* EF2 (EmEF2) available in GenBank (No. 25335462). The forward primer was anchored with *Eco*R I (Takara Biotechnology, Dalian, China) and the reverse primer was anchored with *Hin*d III (Takara Biotechnology, Dalian, China) (Table 1). PCR amplification was executed utilizing 2 × Taq Master Mix (Dye Plus) (Vazyme Biotech, Nanjing, China). The PCR program for the amplification of EmEF2 gene was carried out according to the manufacturer’s protocols. In the meantime, changing the extension time based on the length of EmEF2 gene fragment. The program for EmEF2 was as follows: 95 °C, 3 min; 30 cycles (95 °C, 15 s; 60 °C, 30 s; 72 °C, 149 s); and 72 °C, 5 min. The PCR product of EmEF2 was recovered after *Eco*R I and *Hin*d III digestion in 10 × K Buffer (Takara Biotechnology, Dalian, China) at 37 °C and was subsequently inserted into the pET-32a (Invitrogen Biotechnology, Carlsbad, CA, USA). The constructed pET-32a-EmEF2 was subjected to validation through restriction enzyme digestion and sequence analysis. The complete open reading frame (ORF) of EmEF2 was aligned in the GenBank databases using the Basic Local Alignment Search Tool. Antigenicity analysis of the EmEF2 was performed by Protean of DNAStar software (Version 11.0, DNASTAR Inc., Madison, WI, USA).

### 2.3. Expression of Recombinant Protein EmEF2 and Western Blot Analysis 

pET-32a-EmEF2 was transformed into ampicillin-resistant *Escherichia coli* (*E. coli*) BL21 (DE3) (Vazyme Biotech, Nanjing, China) to express the recombinant protein EmEF2 (rEmEF2). The rEmEF2 was purified using a protein affinity chromatography column (His-Trap™ FF, Cytiva, Marlborough, MA, USA). Subsequently, the purification of rEmEF2 was verified through SDS-PAGE analysis. According to the manufacturer’s protocols, the concentration of purified rEmEF2 was measured using the BCA Protein Assay Kit (Beyotime, Shanghai, China). The concentration of rEmEF2 was 100 µg/mL and the rEmEF2 was used for the following Western blot analysis. Then, the rEmEF2 was 400 µg/mL and used in the subsequent determination of the immune responses and experimental assessment of the protective efficacy of rEmEF2. In addition, the rEmEF2 was cryogenically stored by freezing individual tubes at −80 °C.

The presence of the rEmEF2 was determined through a Western blot assay, using *E. maxima* chicken antiserum, non-infected chicken serum or His-tag monoclonal antibody (Proteintech, Wuhan, China) as primary antibodies, respectively. Here is a concise overview of the procedure: After conducting the SDS-PAGE assay, the purified rEmEF2 was transferred onto a polyvinylidene fluoride (PVDF) membrane (Millipore, Billerica, MA, USA). Subsequently, the PVDF membranes were incubated with *E. maxima* chicken antiserum (1:100 dilution), non-infected chicken serum (1:100 dilution) or His-tag monoclonal antibody (1:200 dilution) for 1 h at 37 °C, respectively. The non-infected chicken serum was regarded as the negative control. After the PVDF membranes were separately incubated with goat anti-chicken IgY H&L (HRP) (1:20,000 dilution, Abcam, Cambridge, UK) or goat anti-mouse IgG H&L (HRP) (1:10,000 dilution, Abcam, Cambridge, UK) for 45 min at 37 °C. The detection of bound antibodies was achieved by initiating a color development process, utilizing an HRP-DAB substrate chromogenic kit (Tiangen, Beijing, China).

### 2.4. Determination of the Immune Responses Induced by rEmEF2 in Chickens 

**Figure 1:**
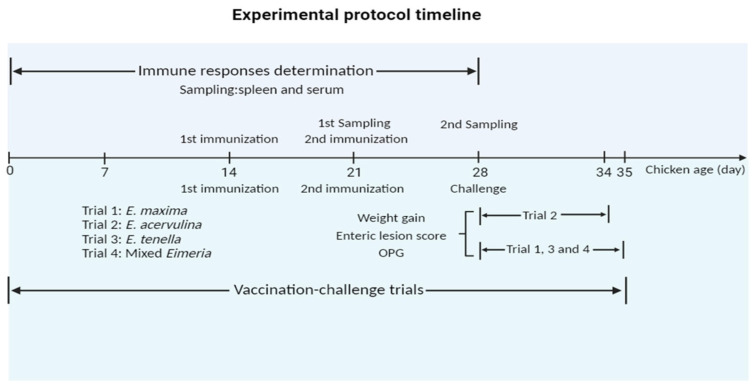
Experimental protocol timeline. The chickens received the first and second vaccinations at 14 and 21 days of age, respectively. In the timeline for the determination of immune responses induced by immunized chickens, spleen lymphocytes and serum samples were collected from immunized chickens at 21 days old and 28 days old, respectively. In timeline of experimental assessment of protective efficacy of rEmEF2 against three *Eimeria*, data collection for *E. acervulina* groups (Trial 2) occurred on the sixth day after the challenge (34 days old), while data collection for other *Eimeria* species (trial 1, 3 and 4) was conducted on the seventh day (35 days old). The collected data included weight gain, enteric lesion score, and OPG. The figure was created in BioRender.com (https://www.biorender.com/ accessed on 2 December 2023).

Chickens (14-day-old) were randomly allocated into three groups, and each group comprised six chickens and underwent the primary immunization. Among these groups, two control groups including a PBS control and a pET-32a tag protein control were intramuscularly injected in the leg as follows: PBS and 200 µg of pET-32a tag protein, respectively. In parallel, the experimental group received intramuscular injections of 200 µg of rEmEF2 in the leg. Following the primary immunization, the secondary immunization was administered after a 7-day interval, and the injection dose of the secondary immunization was the same as the primary immunization. The timeline for the determination of immune responses induced by immunized chickens is shown in Figure 1.

On the 7th day following each vaccination, spleen lymphocytes were gathered in three chickens which were stochastically selected in each group. The spleens were meticulously ground in 5 mL of PBS and filtered with cell strainers. The filtrate containing splenocytes was added to the lymphocyte separation solution (TBDscience, Tianjin, China) and centrifuged at 500× *g* for 20 min, and the lymphocytes located in the middle layer were extracted. The lymphocytes were analyzed using the CD4^+^ and CD8^+^ T cell subpopulations. Whereafter, CD3 mouse anti-chicken FITC antibody (Southern Biotechnology Associates, Birmingham, AL, USA), CD4 mouse anti-chicken PE antibody (Southern Biotechnology Associates, Birmingham, AL, USA) and CD8 mouse anti-chicken PE antibody (Southern Biotechnology Associates, Birmingham, AL, USA) were used to detect the T cell subpopulations with a FACS Calibur flow cytometer (BD Biosciences, Franklin Lakes, NJ, USA). In brief, each group’s lymphocytes suspension was adjusted to 1 × 10^6^ cells in 100 µL of PBS. Subsequently, CD3 and CD4 antibody or CD3 and CD8 antibody were used to bind chicken CD3^+^ CD4^+^ or CD3^+^ CD8^+^, and T cells were incubated for 25 min at 4 °C without light following the manufacturer’s protocols. 

To assess the mRNA transcription levels of the IL-4 gene (GenBank No. AJ621735) and IFN-γ gene (GenBank No. Y07922) in immunized chickens, a quantitative real-time PCR (qPCR) assay was conducted, with the GAPDH gene (GenBank No. K01458) serving as internal control. The primer sequences, amplification efficiency (%) and correlation coefficients (r^2^) of GAPDH, IL-4 and IFN-γ for qPCR are based on previously published articles from our lab [16], and the primer sequences are shown in Table 2. On the 7th day following each vaccination, the total RNA from spleen lymphocytes was extracted from each group of three chickens and subsequently reverse-transcribed to cDNA, following previous protocols. The ChamQ^TM^ SYBR qPCR Master Mix kit (Vazyme Biotech, Nanjing, China) was employed as the manufacturer’s instructions by the qPCR assay. The qPCR amplification reaction system contained 10 µL of 2 × ChamQ^TM^ SYBR qPCR Master Mix, 0.4 µL of forward and reverse primers, 2 µL of cDNA and 7.2 µL of RNase-free water. The triplicated samples were set in each qPCR assay. The reaction procedure of the qPCR was as follows: 95 °C, 30 s; 40 cycles (95 °C, 10 s; 60 °C, 30 s). The melt curve stage of qPCR was as follows: 95 °C, 15 s; 60 °C, 1 min; 95 °C, 15 s. The qPCR reaction was performed on an ABI prism 7300 Fast Real-Time PCR System (Applied Biosystems, Carlsbad, CA, USA). The relative quantification of cytokine gene mRNA was determined using the 2^−ΔΔCt^ method for precise quantification based on the methods previously established by Livak and Schmittgen (2001) [37].

Chicken serum samples were collected from three chickens, which were randomly selected on the 7th day following each vaccination in each group. The indirect enzyme-linked immunosorbent assay (ELISA) was conducted to assess the rEmEF2-specific serum IgY antibody levels. In the indirect ELISA procedure, rEmEF2 was diluted to 10 ng/µL, and 200 µL was coated in flat-bottomed 96-well plates (MarxiSorp, Nunc, Waltham, MA, USA) for 16 h at 4 °C. Subsequently, the plates were washed five times with PBST (PBS with 0.05% Tween20) and blocked with 200 µL of PBST containing 5% bovine serum albumin (BSA) (Yifeixue, Nanjing, China) for 2 h at 37 °C. The primary antibody was chicken serum samples (1:50 dilution) for 1 h at 37 °C, while the secondary antibody was goat anti-chicken IgY H&L (HRP) antibody (1:40,000 dilution). Non-infected chicken serum (1:50 dilution) and PBS were used as controls during the analysis. In the end, the color production was detected with 100 µL of 3,3′,5,5′-tetramethylbenzidine (TMB) (Tiangen, Beijing, China) in the dark at RT for 8 min and observed under OD450 absorbance with a microplate reader (Thermo Fisher Scientific, Waltham, MA, USA).

### 2.5. Experimental Assessment of Protective Efficacy of rEmEF2 against Infections by Three Eimeria Species in Chickens

In order to evaluate the protective efficacy of rEmEF2 (Table 3), four immunization challenge trials were carried out. Trial 1, trial 2, trial 3 and trial 4 were performed to evaluate the protective efficacy of rEmEF2 against *E. maxima*, *E. acervulina*, *E. tenella* and mixed *Eimeria*, respectively. Healthy chickens (14-day-old) were randomly divided into thirteen groups based on similar body weights. Among these, four experimental groups received intramuscular injections of 200 µg of rEmEF2 into the leg, with an injection volume of 0.5 mL. The remaining nine groups served as controls and included a non-immunized non-challenged group, four non-immunized challenged groups and four pET-32a tag protein control groups. The pET-32a tag protein control groups were injected with the same dose of pET-32a tag protein as the experimental groups. The subsequent manipulations were performed as follows. When the chickens reached an age of 21 days, the pET-32a tag protein control groups and the rEmEF2 experimental groups were, respectively, given the same dose as the first immunization for the second immunization. All chickens were orally challenged with 1 × 10^5^ *E. maxima* (trial 1), 1 × 10^5^ *E. acervulina* (trial 2), 5 × 10^4^ *E. tenella* (trial 3), or a mixture of these three *Eimeria* species (trial 4), except for the non-immunized non-challenged group, at age of 28 days according to the grouping in Table 3. The timeline of the protocol schemes of the trials is shown in Figure 1. The life cycles of various *Eimeria* species exhibit distinctions, resulting in diverse peak points for fecal oocyst shedding. Specifically, the highest fecal oocyst-shedding point for *E. tenella* and *E. maxima* occurs on the seventh day after challenge, whereas *E. acervulina* reaches its peak on the sixth day post-challenge. The data collection for *E. acervulina* groups occurred on the sixth day after the challenge, while data collection for other *Eimeria* species was performed on the seventh day. The collected data included body weight gain, enteric lesion score, oocyst shedding and the anticoccidial index (ACI) to assess the protective efficacy of the rEmEF2. We calculated the body weight gain of each chicken based on the difference in weight between the challenge time and the slaughter time. Based on the methods previously established by Hodgson (1970) [38], oocyst counts were carried out using a McMaster chamber. A numerical scale ranging from normal to severe (0 to 4) was used to score the lesions in the chicken intestine, as per Johnson and Reid (1970) [39]. The oocyst-shedding decrease ratio was calculated using the formula: (mean oocyst amount of the challenged control group-that of the vaccinated groups)/oocyst amount of control group × 100%. ACI was calculated using the formula: (relative rate of weight gain + survival rate) − (lesion index + oocyst index) (McManus et al., 1968) [40]. An ACI of less than 120 was deemed to indicate no protective efficacy, while an ACI ranging from 120 to less than 160 was categorized as indicative of low-level protective efficacy. ACI values falling within the range of 160 to less than 180 were considered to represent moderate protective efficacy, while an ACI of 180 or higher was regarded as indicative of excellent protective efficacy.

### 2.6. Statistical Analysis

The data were analyzed for normal distribution using SPSS software (Version 27.0.1, SPSS Inc., Chicago, IL, USA). Statistical analysis was conducted using GraphPad Prism software (Version 8.0.2, GraphPad Software Inc., San Diego, CA, USA), and the significance of differences between groups was assessed through a Kruskal–Wallis H test. The data were presented in the format of mean ± standard deviation (S.D.). A significance level of *p* < 0.05 was considered statistically significant, while *p* > 0.05 indicated a lack of significant difference.

## 3. Results

### 3.1. Cloning of EmEF2 and Recombinant Plasmid Construction of pET-32a-EmEF2

**Figure 2:**
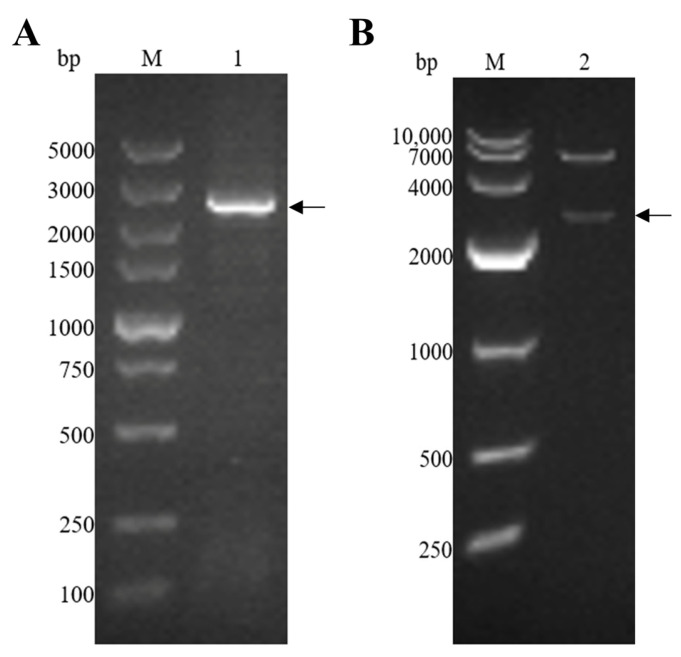
*E. maxima* EF2 (EmEF2) gene cloning and recombinant plasmid construction of pET-32a-EmEF2. (**A**) Cloning of EmEF2 gene from *E. maxima* cDNA. M: DNA marker DL5000. Lane 1: Amplification product of EmEF2 (2499bp). (**B**) Restriction enzyme digestion identification of pET-32a–EmEF2. M: DNA marker DL10000. Lane 2: The pET-32a-EmEF2 was identified by *Eco*R I and *Hin*d III restriction enzyme digestion, resulting in the pET-32a linearized plasmid fragment and the EmEF2.

The EmEF2 was successfully amplified using *E. maxima* cDNA, as previously described. As shown in Figure 2A, the result of the agarose gel electrophoresis showed that the band had a size of 2499 bp, which corresponds to the molecular weight of EmEF2 (Figure 2A, lane 1). Subsequently, the EmEF2 gene was ligated with a prokaryotic expression plasmid: the pET-32a. The recombinant plasmid of pET-32a-EmEF2 was constructed. As shown in Figure 2B, after restriction enzyme digestion (*Eco*R I and *Hin*d III) of the constructed pET-32a-EmEF2, the results showed that the pET-32a linearized plasmid fragment and the target band of 2499 bp were observed, which were consistent with the expected size of EmEF2 (Figure 2B, lane 2). In addition, the sequence analysis confirmed that EmEF2 shared 100% identity with the sequence of *E. maxima* EF2 available in GenBank (No. 25335462). DNAStar Protean analysis revealed that EmEF2 is immunogenic.

### 3.2. Purification of Recombinant Protein EmEF2 and Western Blot Analysis

**Figure 3:**
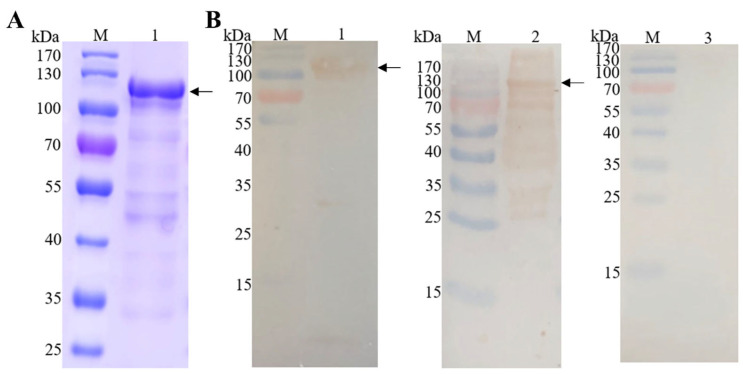
Purification of recombinant protein EmEF2 (rEmEF2) and Western blot analysis. (**A**) SDS-PAGE analysis of rEmEF2 purification (110 kDa). M: protein mid-molecular-weight marker. Lane 1: The rEmEF2 was purified via protein affinity chromatography column (110 kDa). (**B**) Western blot analysis of rEmEF2 (110 kDa). M: protein mid-molecular-weight marker. Lane 1: The purified rEmEF2 was recognized by the His-tag monoclonal antibody as primary antibody and goat anti-mouse IgG H&L (HRP) as secondary antibody. Lane 2: The purified rEmEF2 was recognized by the *E. maxima* chicken antiserum as primary antibody and goat anti-chicken IgY H&L (HRP) as secondary antibody. Lane 3: The non-infected chicken serum was primary antibody and served as negative control, while the secondary antibody was goat anti-chicken IgY H&L (HRP).

The recombinant protein EmEF2 (rEmEF2) was analyzed through SDS-PAGE and Western blot analysis. As shown in Figure 3A, the rEmEF2 was purified via a protein affinity chromatography column; a band appeared at 110 kDa that was consistent with the expected size of rEmEF2 (Figure 3A, lane 1). Furthermore, Western blot analysis confirmed that the purified rEmEF2 was recognized by the His-tag monoclonal antibody (Figure 3B, lane 1) and *E. maxima* chicken antiserum (Figure 3B, lane 2). Meanwhile, the rEmEF2 was not recognized by the negative chicken serum (Figure 3B, lane 3).

### 3.3. The Evaluation of Immune Responses Induced by rEmEF2 in Chickens

**Figure 4:**
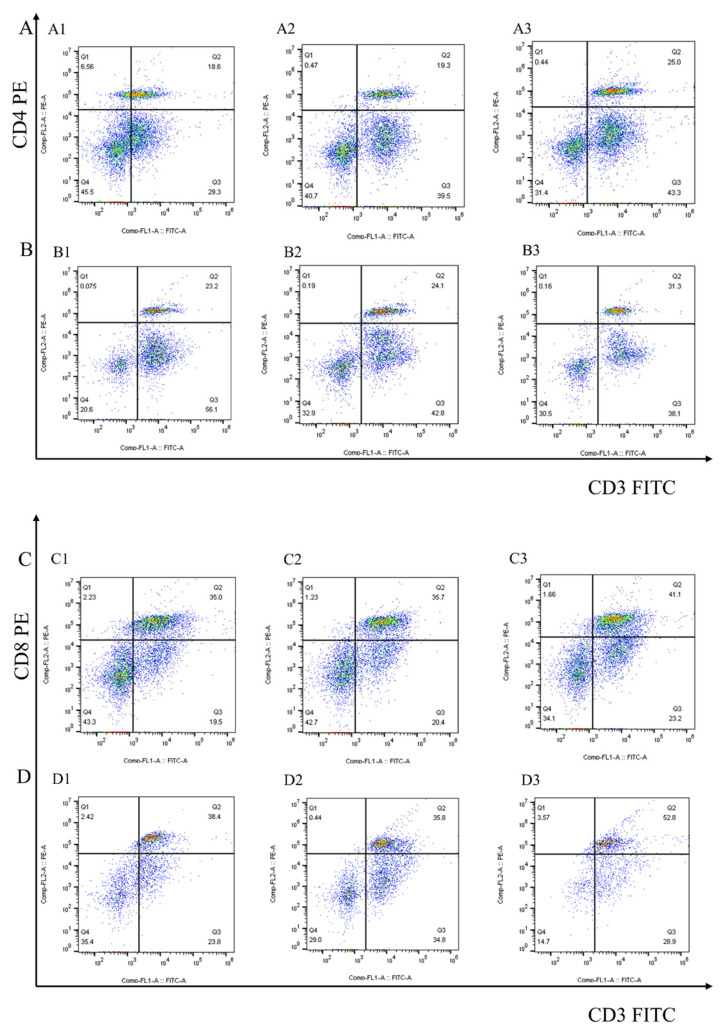
The proportion of the T cell subpopulation in chickens immunized with PBS, pET-32a tag protein and rEmEF2 was determined by flow cytometry 7 days after the first and second immunization. (**A**) Detection of CD3^+^CD4^+^ T lymphocytes in immunized chickens 7 days after the first immunization. (**B**) Detection of CD3^+^CD4^+^ T lymphocytes in immunized chickens 7 days after the second immunization. (**C**) Detection of CD3^+^CD8^+^ T lymphocytes in immunized chickens 7 days after the first immunization. (**D**) Detection of CD3^+^CD8^+^ T lymphocytes in immunized chickens 7 days after the second immunization. 1: PBS control group. 2: pET-32a tag protein control group. 3: rEmEF2 group.

**Figure 5:**
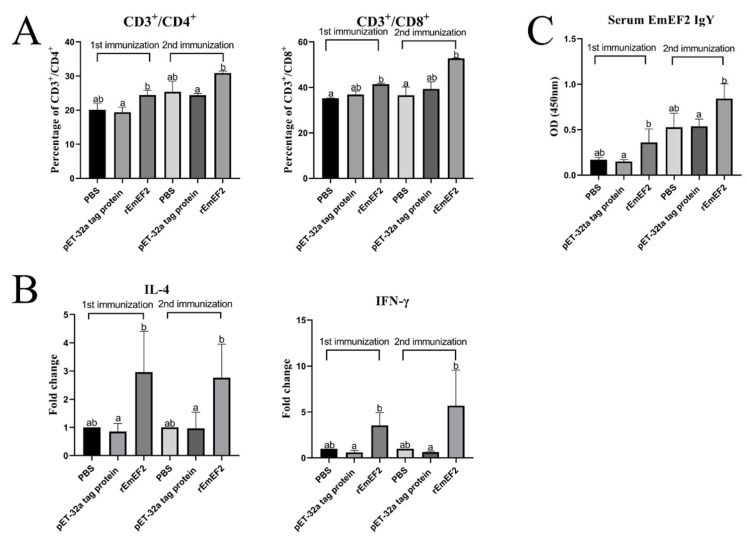
The immune responses induced in immunized chickens 7 days after the first and second immunization. Chickens received the first and second immunizations when aged 14 and 28 days old, respectively. Spleen lymphocytes and serum samples were collected from chickens in each group 7 days after each immunization. The collected spleen lymphocytes were analyzed for changes in T cell subpopulation and cytokine mRNA transcription. Serum samples were analyzed for changes in rEmEF2-specific serum IgY. Significant difference (*p* < 0.05) between data was annotated with different letters. No significant difference (*p* > 0.05) between data was annotated with the same letter. (**A**) Percentage of T cell subpopulation in the spleen of chickens immunized with PBS, pET-32a tag protein and rEmEF2. Left: change in CD4^+^ T lymphocytes. Right: change in CD8^+^ T lymphocytes. (**B**) Change in the mRNA transcription level of cytokine genes in spleen lymphocytes of chickens immunized with PBS, pET-32a tag protein and rEmEF2. Left: change in IL-4. Right: change in IFN-γ. (**C**) Change in rEmEF2-specific serum IgY induced by immunized chicken serum samples 7 days after the first and second immunization.

After 7 days of the first and second immunization with rEmEF2, flow cytometry was used to evaluate the proportion of CD4^+^ and CD8^+^ T lymphocytes in immunized chickens. The results are shown in Figure 4 and Figure 5A. Remarkably, the proportion of CD4^+^ T lymphocytes increased after immunization with rEmEF2 (Figure 5A), when compared to the pET-32a tag protein control group (*p* < 0.05). The proportion of CD8^+^ T lymphocytes increased after immunization with rEmEF2 (Figure 5A), when compared to the PBS control group (*p* < 0.05). Notably, among the PBS control group and the pET-32a tag protein control group, these control groups showed no statistically significant differences in the proportions of CD4^+^ and CD8^+^ T lymphocytes (*p* > 0.05).

After 7 days of both the first and second immunization with the rEmEF2, the mRNA transcription levels of IL-4 and IFN-γ in immunized chickens were assessed by qPCR analysis. As shown in Figure 5B, the qPCR results indicated that the mRNA transcription levels of cytokine IL-4 and IFN-γ were increased in the rEmEF2 immunization group compared to the pET-32a tag protein control group (*p* < 0.05). Notably, there were no statistically significant differences in the mRNA transcription levels of cytokines among the control groups (*p* > 0.05).

The rEmEF2-specific IgY levels in the serum of chickens immunized with rEmEF2 were detected using indirect ELISA assays. As exhibited in Figure 5C, the rEmEF2-specific IgY antibody levels in the rEmEF2-immunized group were higher than the pET-32a tag protein control group seven days after the first and second immunization (*p* < 0.05). Notably, there was no significant difference in IgY antibody levels between the control groups (*p* > 0.05).

### 3.4. Protective Efficacy of rEmEF2 Vaccines against E. maxima, E. acervulina, E. tenella and Mixed Eimeria

The protective efficacy of rEmEF2 was assessed based on parameters such as relative weight gain, oocyst decreased ratio, lesion score and ACI. The results of all data are shown in Table 3. Chickens immunized with rEmEF2 were then orally challenged with *E. maxima*, *E. acervulina*, *E. tenella* and mixed *Eimeria*, resulting in relative body weight gains, which were 87.06%, 97.58%, 71.87% and 57.56% (Table 3), respectively. Notably, the weight gain of the experimental groups (immunized with rEmEF2) was significantly higher than that of the control groups (non-immunized challenged and pET-32a tag protein control) (*p* < 0.05). These findings clearly indicate that immunization with rEmEF2 significantly mitigated the weight gain loss caused by *Eimeria* infection.

The reduction in mean enteric lesion scores and Oocysts Per Gram (OPG) in the rEmEF2-immunized groups were found to be significantly lower compared to the non-immunized challenged groups, as well as the pET-32a tag protein control groups (*p* < 0.05). Chickens immunized with rEmEF2 were then orally challenged with *E. maxima*, *E. acervulina*, *E. tenella* and mixed *Eimeria*, resulting in decreased oocyst ratios, which were 70.84%, 83.47%, 94.56% and 92.86% (Table 3), respectively. These findings show that immunization with rEmEF2 alleviated the enteric lesions and reduced oocyst output in the *Eimeria*-infected chickens. 

In the case of rEmEF2-immunized groups, challenges with *E. maxima* and *E. acervulina* resulted in ACI scores of 166.35 and 185.08 (Table 3), signifying moderate-to-excellent protective efficacy. However, challenges involving *E. tenella* and mixed *Eimeria* species yielded ACI scores of 144.01 and 127.94, indicating a low level of protective efficacy. 

## 4. Discussion

*Eimeria* species are an obligate intracellular parasitic protozoan and can cause avian coccidiosis clinically, which seriously affects the health, efficiency and sustainable development of the domestic poultry industry and brings tremendous and irreversible loss worldwide [1,41,42]. The data report on avian coccidiosis shows that the prevalence range of *Eimeria* spp. is widespread and the infection rate is high [41]. Recent reports estimate that avian coccidiosis in poultry is responsible for a global cost of around GBP 10.4 billion [3,4,43]. Anticoccidial drugs and live vaccines have been used strictly in clinical practice because of their various drawbacks, such as drug resistance and residues, the high cost of producing live vaccines, the inconvenient transportation of live vaccines and so on. In existing research on the prevention of avian coccidiosis, neotype vaccines have been revealed as a potential and prospective strategy against *Eimeria* species that are short of the shortcomings of anticoccidial drugs and traditional vaccines [44,45]. Nevertheless, the search for feasible vaccine candidate antigens has always been a formidable and hard task in the development of subunit, DNA and live vector vaccines. In *Eimeria* species, various antigens have been identified as antigen candidates for subunit vaccines and DNA vaccines, inducing immune responses and providing immune protection [16,18,19,25]. In this study, an *Eimeria* common antigen of EF2 was used to construct neotype anticoccidial vaccines: recombinant subunit vaccine (rEmEF2). The vaccination challenge trial showed that the vaccines constructed in the study could provide partial protection against infection by single or mixed *Eimeria* species. The result indicates that EmEF2, a common antigen, is an effective candidate antigen for the substantial development of neotype vaccines against mixed infection by *Eimeria* species.

The majority of clinical cases of chicken coccidiosis result from mixed infections [2,46,47,48]. Consequently, commercially available traditional live anticoccidial vaccines are typically multivalent. The same holds true for neotype anticoccidial vaccines, as monovalent vaccines may not adequately address the clinical requirements [5,49]. In recent times, a few strategies have been employed to develop multivalent vaccines for chicken coccidiosis. One approach involves the use of a cocktail of antigens derived from different *Eimeria* species, which has demonstrated promising immune protection [50,51]. Another strategy entails the design of multiepitope DNA vaccines comprising multiple genes sourced from various chicken *Eimeria* species, displaying notable protective efficacy against multiple *Eimeria* strains [45,52]. In this specific study, we chose the common *Eimeria* antigen EF2 as a candidate antigen and observed that vaccination with EmEF2 provided partial protection against infection by three *Eimeria* species when administered recombinant subunit vaccine (rEmEF2). These findings present an additional avenue for the development of effective and safe multivalent anticoccidial vaccines.

In the present study, we investigated the immune responses elicited by vaccination with the common *Eimeria* antigen EF2. Cellular immunity has been recognized as a dominant player in the defense against *Eimeria* infection, with T cells and their secreted cytokines playing pivotal roles [53,54,55]. Notably, CD4^+^ T cells and CD8^+^ T cells have demonstrated significant involvement in combating avian coccidia infections [4]. The IFN-γ as a Th1 cytokine exerts crucial early anticoccidial effects [56,57,58]. In this study, the proportion of CD4^+^ and CD8^+^ T lymphocytes showed an increase after the first and second immunization with the rEmEF2, indicating that T cells play a positive role in avian coccidiosis infection. Specifically, IFN-γ mRNA transcript levels increased seven days after the first and second immunization with rEmEF2. These results suggest that EmEF2 positively contributes to resistance against coccidiosis infection, with the observed changes in T lymphocytes and cytokine levels indicative of a robust cell-mediated immune response. In this study, the mRNA transcription level of IL-4 was also increased by vaccination with the rEmEF2. These results align with previous reports [59]. The role of humoral immune responses has been debated in *Eimeria* infection. Recent studies have demonstrated that maternal or passive immunization can provide protective antibodies, impeding the growth and development of *Eimeria* species and safeguarding chick offspring [60,61]. In our study, vaccination with rEmEF2 led to an increase in IgY antibody levels following both the initial and booster immunization. These results lend support to the notion that IgY contributes to the anticoccidial immune response. Overall, EmEF2 administered as a recombinant subunit vaccine induced significant cellular and humoral immune responses, underscoring its pivotal role in immune protection.

EF2 has been shown to offer critical protection against various parasites. For example, in *Leishmania* challenge experiments, EF2 induced elevated levels of IL-12 and IFN-γ, mediated Th1 immune responses, and significantly increased IgG2 antibody levels, resulting in 65% protection in hamsters [33]. Additionally, the localization of *Eimeria tenella* EF2 (EtEF2) in second-generation merozoites has been determined, with increased expression levels, leading to the inhibition of partial invasion-related proteins following diclazuril treatment [59,62]. These results underscore the significant prospects and potential of EF2 as a vaccine candidate antigen given its high homology across species and its demonstrated capacity to confer significant immune protection.

In this study, we used the whole protein of EmEF2 to construct an anticoccidial vaccine. However, developing peptide vaccines could be considered for controlling chicken coccidiosis. Some researchers have proposed that peptide-based vaccines are composed of immunogenic epitopes of various antigens to generate highly specific immune responses [63,64,65,66,67]. Since cellular immunity plays a crucial role against avian coccidiosis, selecting peptides of T cell epitopes as vaccine candidates for development may effectively control the incidence of avian coccidiosis [68,69,70,71,72]. Therefore, T cell epitopes of several common antigens of chicken coccidia identified in our previous study, including EF2, can be selected for the construction of a multivalent epitope vaccine, which can be targeted to increase the level of cellular immunity induced by the vaccine and thus improve its protective efficacy. We will be conducting such studies in the future.

Immunization with EmEF2 in chickens effectively ameliorated enteric lesions, reduced weight loss, and diminished oocyst output in chickens afflicted with single or mixed *Eimeria* species infections. Nevertheless, the scope for enhancing their protective efficacy remains. For example, the incorporation of cytokines such as IL-2 or IFN-γ as adjuvants [73,74,75], or their direct inclusion into prokaryotic recombinant plasmids, coupled with potential adjustments to the dosage of EmEF2 vaccines, may serve to augment the immunoprotective potency of EmEF2 vaccines. 

Since the intramuscular injection route could provide consistency in systemic immune responses [76], it is commonly employed in studies involving new-generation anticoccidial vaccines. Nasri et al. reported intramuscular injection as the most prevalent administration route for new-generation anticoccidial vaccines (n  =  43 studies) [77]. In their report, they took a meta-analysis of the immunization routes used in immunization challenge trials which evaluated the protective efficacy of new-generation anticoccidial vaccine candidates against *Eimeria* infection in chickens; they found that out of 63 studies, 43 utilized intramuscular injection. Notably, 25 studies employed intramuscular injection for delivering subunit vaccines, indicating its frequent use as a route for administering anticoccidial subunit vaccines in chickens. Therefore, we also employed the intramuscular route in this study, resulting in effective immunoprotection. However, intramuscular injection inevitably causes stress to immune animals, leading to potential deviations in immune responses. Non-injection immunization (e.g., via eye and nasal drops) [78,79,80] is more suitable for large-scale clinical practice than intramuscular immunization. In future studies, we will consider non-injectable vaccination routes to immunize chickens.

## 5. Conclusions

In conclusion, EmEF2 is highly immunogenic, elicits immune responses and is able to provide partial protection against both single and mixed *Eimeria* species infections, suggesting that EmEF2 is a promising candidate antigen and offers a hopeful prospect for the development of anticoccidial vaccines to prevent *Eimeria* infection.

## Figures and Tables

**Table 1:** Primers of *E. maxima* EF2 (EmEF2).

Gene	Primer (5′-3′)	Accession No.
EmEF2	Forward: CCGGAATTCATGGTGAATTTTTCAGTGGATC	25335462
	Reverse: CCCAAGCTTTTACAGCTTGTCGTAGTAGTGGTCG	

**Table 2:** Primer sequences of GAPDH, IL-4 and IFN-γ.

RNA Target	Primer Sequence (5′-3′)	Accession No.
GAPDH	Forward: GGTGGTGCTAAGCGTGTTAT	K01458
	Reverse: ACCTCTGTCATCTCTCCACA	
IL-4	Forward: ACCCAGGGCATCCAGAAG	AJ621735
	Reverse: CAGTGCCGGCAAGAAGTT	
IFN-γ	Forward: GGTGGTGCTAAGCGTGTTAT	Y07922
	Reverse: ACCTCTGTCATCTCTCCACA	

**Table 3:** Protective efficacy of rEmEF2 vaccines against the challenge infections by *E. maxima*, *E. acervulina*, *E. tenella* and mixed *Eimeria*.

Trials	Groups	N	Initial Body Weight (g)	Weight Gain (g)	Relative Body Weight Gain (%)	Mean Enteric Lesion Scores	OPG (×10^5^)	Oocyst Decreased Ratio (%)	Anticoccidial Index (ACI)
1	Non-immunized non-challenged	14	138.25 ± 6.96 ^a^	103.83 ± 36.48 ^b^	100.00	0 ± 0 ^a^	0 ± 0 ^a^	100.00	200
	Non-immunized challenged	9	135.32 ± 10.01 ^a^	26.82 ± 16.00 ^a^	25.13	3.78 ± 0.44 ^c^	1.79 ± 0.985 ^c^	0.00	47.35
	pET-32a tag protein control	12	138.73 ± 7.22 ^a^	43.31 ± 12.22 ^a^	39.99	3.92 ± 0.29 ^c^	2.07 ± 1.05 ^c^	−15.64	60.82
	rEmEF2	14	140.68 ± 4.52 ^a^	94.21 ± 20.06 ^b^	87.06	1.07 ± 0.47 ^b^	0.522 ± 0.257 ^b^	70.84	166.35
2	Non-immunized non-challenged	14	138.25 ± 6.96 ^a^	78.36 ± 34.14 ^b^	100.00	0 ± 0 ^a^	0 ± 0 ^a^	100.00	200
	Non-immunized challenged	15	140.9 ± 4.70 ^a^	52.13 ± 16.2 ^a^	64.16	3.93 ± 0.26 ^b^	10.1 ± 8.22 ^c^	0.00	84.78
	pET-32a tag protein control	14	138.34 ± 6.17 ^a^	52.01 ± 25.14 ^a^	65.47	3.79 ± 0.43 ^b^	6.67 ± 4.79 ^c^	33.96	107.61
	rEmEF2	16	136.76 ± 10.54 ^a^	76.41 ± 21.81 ^b^	97.58	0.75 ± 0.58 ^a^	1.67 ± 1.31 ^b^	83.47	185.08
3	Non-immunized non-challenged	14	138.25 ± 6.96 ^a^	103.83 ± 36.48 ^b^	100.00	0 ± 0 ^a^	0 ± 0 ^a^	100.00	200
	Non-immunized challenged	14	138.51 ± 6.71 ^a^	25.84 ± 36.86 ^a^	24.08	3.86 ± 0.36 ^c^	91.9 ± 64.8 ^c^	0.00	65.51
	pET-32a tag protein control	10	139.65 ± 6.12 ^a^	45.36 ± 14.78 ^a^	43.11	3.80 ± 0.42 ^c^	138 ± 98.2 ^c^	−50.16	65.11
	rEmEF2	14	140.34 ± 3.48 ^a^	78.23 ± 31.54 ^b^	71.87	2.29 ± 1.38 ^b^	5 ± 6.4 ^b^	94.56	144.01
4	Non-immunized non-challenged	14	138.25 ± 6.96 ^ab^	103.83 ± 36.48 ^b^	100.00	0 ± 0 ^a^	0 ± 0 ^a^	100.00	200
	Non-immunized challenged	7	140.06 ± 5.46 ^ab^	0.10 ± 25.34 ^a^	0.10	3.71 ± 0.49 ^c^	36.4 ± 32.9 ^c^	0.00	22.96
	pET-32a tag protein control	15	140.06 ± 6.85 ^b^	−5.41 ± 14.44 ^a^	−5.23	3.93 ± 0.26 ^c^	41.3 ± 32.3 ^c^	−13.46	15.44
	rEmEF2	13	134.88 ± 5.41 ^a^	59.47 ± 22.91 ^b^	57.56	2.46 ± 1.33 ^b^	2.6 ± 1.1 ^b^	92.86	127.94

Notes: (1) trial 1, trial 2, trial 3 and trial 4 were performed to evaluate the protective efficacy of rEmEF2 against *E. maxima*, *E. acervulina*, *E. tenella* and mixed *Eimeria*, respectively. (2) Value = mean ± standard deviation (S.D.). (3) Data comparisons were performed only within the challenged groups of the same *Eimeria* species, not among the challenged groups of different *Eimeria* species. (4) Data collection for the *E. acervulina* groups occurred on the sixth day after the challenge, while data collection for other *Eimeria* species was performed on the seventh day. (5) The mixed *Eimeria* included 1 × 10^5^ *E. maxima*, 1 × 10^5^ *E. acervulina* and 5 × 10^4^ *E. tenella*. (6) Significant difference (*p* < 0.05) between data was annotated with different letters. No significant difference (*p* > 0.05) between data was annotated with the same letter.

## Data Availability

The data presented in this study are available within the article.
